# Contraction of human brain vascular pericytes in response to islet amyloid polypeptide is reversed by pramlintide

**DOI:** 10.1186/s13041-023-01013-1

**Published:** 2023-02-15

**Authors:** Cristina Nuñez-Diaz, Dovilė Pocevičiūtė, Nina Schultz, The Netherlands Brain Bank, Charlotte Welinder, Karl Swärd, Malin Wennström

**Affiliations:** 1 Cognitive Disorder Research Unit, Department of Clinical Sciences Malmö, Lund University, Malmö, Sweden; 2 Netherlands Institute for Neuroscience, Meibergdreef 47, 1105 BA Amsterdam, The Netherlands; 3 Faculty of Medicine, Department of Clinical Sciences, Lund, Mass Spectrometry, Lund University, Lund, Sweden; 4 Department of Experimental Medical Science, Lund University, Lund, Sweden

**Keywords:** Amylin, Vasculopathy, Mural cells, Diabetes, Blood flow

## Abstract

The islet amyloid polypeptide (IAPP), a pancreas-produced peptide, has beneficial functions in its monomeric form. However, IAPP aggregates, related to type 2 diabetes mellitus (T2DM), are toxic not only for the pancreas, but also for the brain. In the latter, IAPP is often found in vessels, where it is highly toxic for pericytes, mural cells that have contractile properties and regulate capillary blood flow. In the current study, we use a microvasculature model, where human brain vascular pericytes (HBVP) are co-cultured together with human cerebral microvascular endothelial cells, to demonstrate that IAPP oligomers (oIAPP) alter the morphology and contractility of HBVP. Contraction and relaxation of HBVP was verified using the vasoconstrictor sphingosine-1-phosphate (S1P) and vasodilator Y27632, where the former increased, and the latter decreased, the number of HBVP with round morphology. Increased number of round HBVP was also seen after oIAPP stimulation, and the effect was reverted by the IAPP analogue pramlintide, Y27632, and the myosin inhibitor blebbistatin. Inhibition of the IAPP receptor with the antagonist AC187 only reverted IAPP effects partially. Finally, we demonstrate by immunostaining of human brain tissue against laminin that individuals with high amount of brain IAPP levels show significantly lower capillary diameter and altered mural cell morphology compared to individuals with low brain IAPP levels. These results indicate that HBVP, in an in vitro model of microvasculature, respond morphologically to vasoconstrictors, dilators, and myosin inhibitors. They also suggest that oIAPP induces contraction of these mural cells and that pramlintide can reverse such contraction.

## Introduction

The islet amyloid polypeptide (IAPP), also known as amylin, is a 37 aa polypeptide expressed by pancreatic islet β-cells, where it is postprandially co-secreted with insulin. In its monomeric form, IAPP physiological functions include delaying gastric emptying, promoting satiation, and reducing postprandial glucagon secretion [1]. However, oligomeric and fibrillar forms of IAPP are related with pathological conditions, such as type 2 diabetes mellitus (T2DM). This disease is characterized by the accumulation of IAPP aggregates in the pancreas [2–4]. Besides, the polypeptide has been linked with Alzheimer’s disease (AD), since IAPP deposits have been found in the brains of AD patients, regardless of T2DM diagnosis. The pancreatic IAPP is known to cross the blood brain barrier (BBB) [5, 6] and under normal conditions bind to amylin receptor in specific brain regions and thereby regulate appetite [1]. However, in AD patients the peptide is found as deposits in the parenchyma, vessel walls, and perivascular space, often co-localizing with amyloid beta (Aβ) [7, 8], which is one of the main pathological hallmarks of AD. This is interesting given that the risk of AD in T2DM patients is strongly linked to vascular complications (e.g., microvascular disease, diabetic foot, cerebrovascular disease, cardiovascular disease) associated with the disease [9]. The significance of vessel-associated IAPP depositions in the brain is not known, but a previous study has shown that IAPP accumulation in brain vessel leads to loss of thight junctions and decreased endothelial cell coverage in rats [10]. In addition, we have demonstrated that oligomeric IAPP (oIAPP), but also IAPP fibrils (although to a lesser extent compared to oIAPP), are highly toxic to cultured primary human brain vascular pericytes (HBVP) [11]. Pericytes are mural cells which enclose the capillaries in the brain and form, together with endothelial cells and astrocytes, the BBB. Pericytes are therefore implicated in BBB permeability, regulation and clearance of debris and toxic substances (e.g., Aβ). Pericytes also play a crucial role in angiogenesis and leukocyte extravasation [12], and regulate capillary blood flow by relaxing and contracting their endothelial tube enwrapping projections [13]. It may thus be that IAPP, via its impact on pericytes, strongly affects Aβ clearance, BBB permeability, and contraction. Indeed, studies have shown that monomeric IAPP and pramlintide (its non-aggregative analog) enhance Aβ clearance from the brain to the blood [14], and an association between BBB permeability and total IAPP levels in cerebrospinal fluid (CSF) has been reported [15]. Whether IAPP interferes with capillary contraction is less explored. Therefore, the purpose of this study is to use an in vitro brain microvasculature model to elucidate whether human oIAPP affects the morphology and contractility of mural cells. We will also investigate the potential link between IAPP and capillary contraction by analyzing capillary diameters and alterations in mural cell morphology in the hippocampal region CA1 of individuals with high and low amount of IAPP in hippocampus.

## Materials and methods

### Cells

Human cerebral microvascular endothelial cells (hCMEC/D3, Millipore, #SCC066) and primary human brain vascular pericytes (HBVP, ScienCell, #1200) were grown in complete endothelial cell growth medium (EGM-2MV, Lonza, #CC-3129) and complete pericyte cell culture medium (PM, ScienCell, #1201), respectively. For monolayer culture, endothelial cells were grown in rat tail collagen I (Gibco, #A1048301) coated 6-well plates and HBVP were cultured in poly-L-lysine (ScienCell #0413) coated 6-well plates, both in humidified air with 5% CO_2_ at 37 °C until 70–80% confluent.

### Proteomics

The HBVP were evaluated prior to experiment using proteomics. The HBVP were grown in poly-L-lysine (ScienCell #0413) coated 12-well plates in 3 replicates and thereafter lysed in RIPA buffer (Sigma Aldrich #R0278). For proteomics, 100 µL was reduced with dithiotreitol and alkylated with iodoacetamide followed by protein precipitation with ice cold ethanol overnight at − 20 °C. The samples were centrifuged and the pellets were resuspended in 100 mM ammonium bicarbonate and protein concenteration were determined. Samples, 30 µg, were digested with trypsin overnight. The generated peptides were injected into liquid chromatography–tandem mass spectrometry (LC–MS/MS). The generated MS/MS spectra were searched using Proteome Discoverer 2.5 (Thermo Fisher Scientific) against UniProt Human (UP000005640). The precursor tolerance and fragment tolerance were set to 15 ppm and 0.05 Da, respectively. Trypsin was selected as enzyme, methionine oxidation and deamidation of aspargine and glutamine were treated as dynamic modification, and carbamidomethylation of cysteine as a fixed modification. The dataset showed that the HBVP expressed mural cell markers such as PDGFβ receptor and NG2, but not PDGFα receptor nor Dcn. Proteins involved in the contractility machinery, including transgelin, myosin regulatory light chain 9, tropomyosin beta chain, calponin 1, myosin 9 and myosin 10 were also found.

### Stimuli for cell culture

IAPP oligomers were prepared based on a previously published protocol [16], which yields IAPP bands between 4 and 25 kDa on a western blot [11]. Briefly, lyophilized synthetic human IAPP_1-37_ (AlexoTech AB #AI-452-10) was solubilized in 10 mM NaOH (pH 11). For adjusting the pH to 7, phosphate buffer was added to a final concentration of 100 µM. For oligomer formation, IAPP was incubated with agitation for 20 min at room temperature. Pramlintide acetate salt (Pram) (Sigma Aldrich #SML2523) Y27632 (Sigma Aldrich, #SCM075), and AC187 (Tocris, #3419) were resuspended in bi-distilled H_2_O. Sphingosine 1 phosphate (S1P) (Sigma Aldrich, #73914-1MG) was solubilized in NaOH 0.3 M to a stock concentration of 1 mM. Blebbistatin (Blebb) (Tocris, #1760) was resuspended in dimethyl sulfoxide to a concentration of 100 mM.

### Matrigel model

For the Matrigel model, glass bottom angiogenesis µ-slides (Ibidi #81507) were coated with a layer of Matrigel basement membrane matrix (10 mg/mL) (Corning # 354234) and incubated for 30 min at 37 °C for polymerization. The cells in monolayer culture were 70–80% confluent, in passages 4–6 (HBVP) and 31–33 (hCMEC/D3), and with a doubling time of approximately 48 h. HBVP were labelled with CellTracker™ Green CMFDA dye (Invitrogen, #C7025) prior to trypsinization, following the manufacturer’s instructions. Afterwards, hCMEC/D3 and HBVP were co-seeded on top of the Matrigel layer at a density of 200,000 and 100,000 cells/mL, respectively, using 50 µL of medium (EGM-2MV 10% FBS) per well. Three hours after seeding, when the cells have already formed capillary-like structures (CLS), stimulation was performed with IAPP 10 µM, sphingosine 1 phosphate 0.1 µM, Y27632 1 µM (alone or combined with IAPP), AC187 10 µM (combined with IAPP), pramlintide 1 µM (alone or combined with IAPP), blebbistatin 1 µM (combined with IAPP), and the corresponding vehicle conditions. The model was stimulated with the different conditions for 12 h. After stimulation, the microvascular model was stained with trypan blue (Bio-Rad, #1450013) for cell death analysis. After 12 h of stimulation, two randomly chosen fields of view of each well were captured with an Olympus BX41 light microscope with the 10 × objective (6 replicates per condition, 2 fields per replicate). To avoid biases, only after the fields were selected in the bright field microscopy, the images of fluorescent pericytes were captured. The numbers of total HBVP and round HBVP per arm were quantified in the fluorescent images using ImageJ software, where a threshold was set for the detection of green labeled HBVP, as well as a circularity filter of 0.8–1.0 (where 1.0 is a perfect circle).

### Individuals included in the study

Human hippocampal samples were provided by the Netherlands Brain Bank (NBB), where neuropathological assessment of Aβ, neurofibrillary tangles and neuritic plaques were performed according to ABC staging [17]. NBB also evaluated Lewy bodies (LB) stage according to Braak et al. [18]. Sex, age, neuropathological assessment (ABC, LB), IAPP levels, diagnosis of T2DM, postmortem delay, and cause of death of the individuals are shown in Table 1. The individuals included in the study showed little or no Aβ deposition in hippocampus to avoid interference of this peptide with vessel diameter. The levels of IAPP in the hippocampus of the individuals were measured previously and described by Schultz et al. [19]. The individuals were divided in two groups based on the mean value (10 pg/mL) of their total IAPP levels (soluble and insoluble), (n = 4) cases with IAPP levels below the mean value (IAPP low) (4.83–6.05 pg/mL) and (n = 4) cases with IAPP levels above the mean value (IAPP high) (13.63–15.93 pg/mL). In all cases, informed consent for using brain tissue and clinical data for research was obtained from the patients or their closest relatives in accordance with the International Declaration of Helsinki and the Code of Conduct for Brain Banking. The medical ethics committee of VU Amsterdam approved the tissue collection procedures and the Swedish Ethical Review Authority approved the study. All data were analyzed anonymously.

**Table 1:** Clinical data of individuals included in the study

Sex	Age (years)	ABC^1^ staging	LB^2^ (0–6)	T2DM	IAPP levels (pg/ml)	Postmortem delay (h:min)	Cause of death
M	70	A0B1C0	3	No	4.83	6:20	Pneumonia + cardiogenic shock
M	63	A0B1C0	0	Yes	5.18	5:00	Unclear, possible infection
M	75	A2B1C1*	0	No	5.70	7:10	Cardiac arrest with COPD
F	60	A0B0C0	0	No	6.05	8:10	Breast cancer with metastasis
F	92	A0B2C0	1	No	13.63	6:35	Heart failure
F	68	A0B0C0	0	Yes	13.67	4:30	Euthanasia
M	102	A1B2C1*	0	No	14.54	5:00	Ileus
F	69	A0B0C0	0	Yes	15.93	5:25	Cachexia and infection

^1^ABC Montine staging of Alzheimer’s disease. A0-3: stain for Aβ/amyloid plaques. B0-3: stain for neurofibrillary tangles. C0-3: stain for neuritic plaques. ^2^LB: 0–6 Lewy Body staging according to Braak. T2DM: Type 2 Diabetes Mellitus. COPD: chronic obstructive pulmonary disease *0–2 diffuse amyloid plaques found in CA1

### Immunostaining and vessel quantification

The hippocampal tissue was fixed in 4% paraformaldehyde for 4 h and then cryoprotected in phosphate-buffered saline with 30% sucrose until it sank. The tissue was sliced into sections of 40 µm thickness using a microtome (Leica SM 2010R). The sections were stored free-floating in cryoprotectant antifreeze solution at − 20 °C. In order to analyze the diameter of brain vessels and mural cell morphology, the sections were stained against laminin α5, expressed by multiple mural cell types and vascular cells, including pericytes and endothelial cells. First, the sections were incubated in quenching solution (3% H_2_O_2_, 10% methanol) for 30 min, followed by Impress reagent kit blocking solution (Vector Laboratories #MP-7402) for 1 h at room temperature and then incubation with mouse anti-laminin (clone 4C7, Dako) in blocking solution overnight at 4 °C. Afterwards, the sections were incubated in Ig Impress reagent kit secondary anti-mouse antibody (Vector Laboratories #MP-7402) at room temperature for 2 h, and then developed for 2 min in 0.25 mg/mL diaminobenzidine and 0.012% H_2_O_2_. The sections were mounted with DPX (Sigma Aldrich, #06522) and pictures of CA1 hippocampal region (stratum lacunosum-moleculare) were captured with an Olympus BX41 light microscope with 20 × objective (two sections of each individual, 20 fields in total). The diameter of total laminin-enclosed capillaries and the diameter of laminin-enclosed capillaries near mural cells were measured by a blinded observer using ImageJ software. Differences in laminin-enclosed mural cell morphology were analyzed by measuring the height of the laminin-enclosed mural cell bodies (distance between the highest point of mural cells and the capillary) found in the images using SenseCell software (Olympus). More than 70 mural cells per individual were analyzed.

### Statistics

Statistical analysis was performed using Prism software (version 9.2.0, GraphPad). For normal distribution assessment, Kolmogorov–Smirnov test was performed. Our data was normally distributed, therefore, we performed independent-samples t-test or one-way ANOVA followed by Dunnett or Tukey test. Results are represented as means ± standard deviations. A value of p < 0.05 was considered significant.

## Results

### Analysis of the effects of IAPP stimulation in vitro

**Fig. 1:**
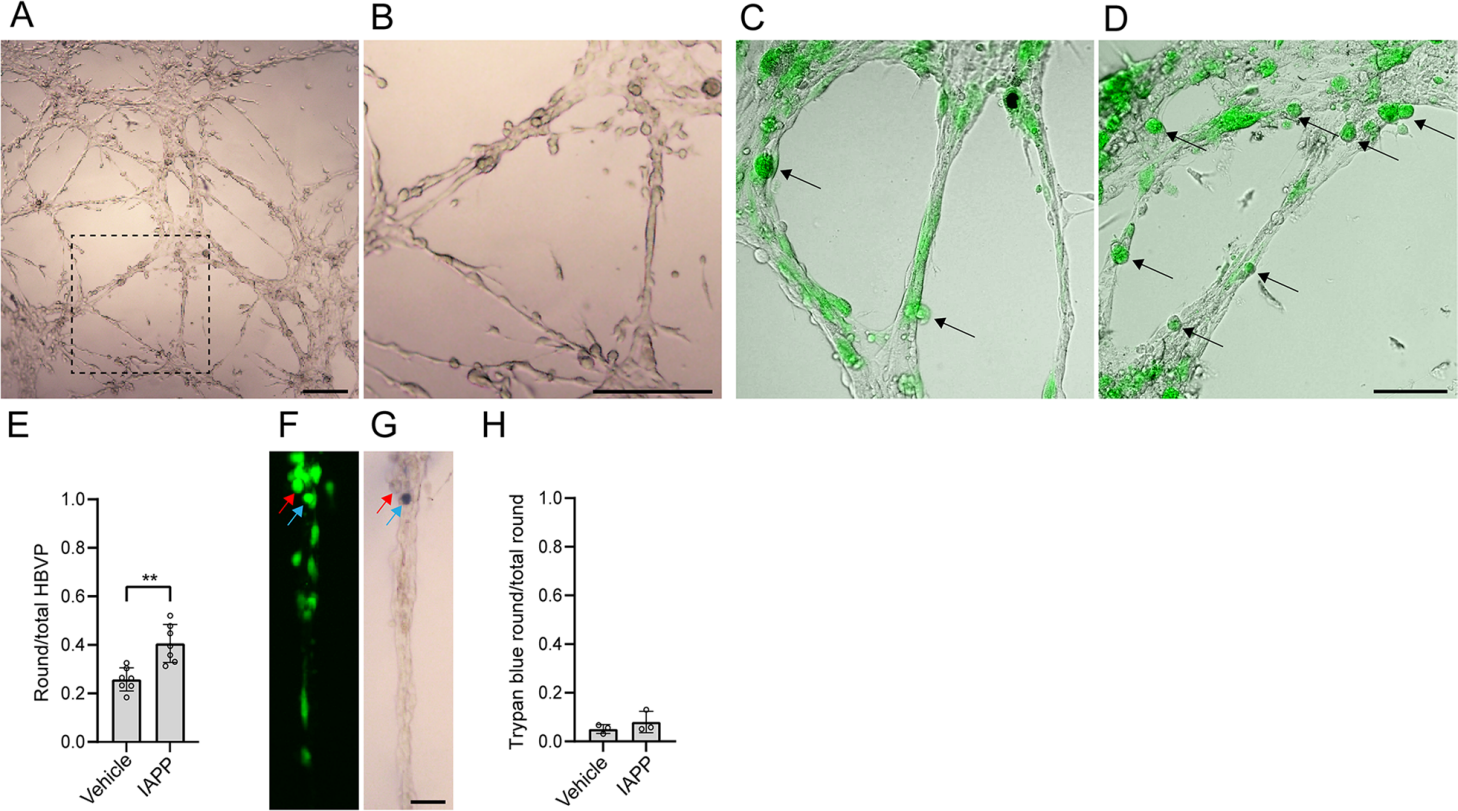
Effects of oIAPP on HBVP in the microvascular model. **A** shows capillary-like structures (CLS) formed by HBVP and endothelial cells co-cultured on a gel matrix. Higher magnification of **(A)** is seen in **(B)**. Scale bars in **(A)** and **(B)**: 200 µm. In **(C)** and **(D)** CLS after treatment with vehicle **(C)** and oIAPP **(D)**, with higher proportion of round-shaped HBVP (black arrows) in the latter. Scale bar in **(C)** and **(D)**: 100 µm. Quantification of the proportion of round-shaped HBVP per CLS after stimulation is seen in **(E)**. Images in **(F)** and **(G)** show CLS stained with green Cell Tracker **(F)** and trypan blue **(G)**, where the red arrow indicates a round HBVP negative for trypan blue and the blue arrow indicates a trypan blue-positive round HBVP. Scale bar in **(F)** and **(G)**: 50 µm. The quantification of the proportion of trypan blue-positive HBVP among total round HBVP per CLS after stimulation is shown in **(H)**. Images **(A)** and **(B)** were acquired with the 4 × objective, images in **(C)**, **(D)**, **(F)** and **(G)** were acquired with the 10 × objective. Experiments in **(E)** and **(H)** were performed in 6 replicates and data was analyzed using student t-test. Each point represent the mean ± SD. Significant difference at **p < 0.01

As described previously [20, 21], the co-culturing of endothelial cells and HBVP resulted in self-assembled cell clusters connected by branches, resembling a network of capillaries without lumen (capillary-like structures, CLS) (Fig. 1A, B). The CLS consisted of HBVPs tightly associated with the elongated endothelial cells. Many of HBVP were localized in the peripheral part along the branch (Fig. 1C, D). After stimulating the CLS with oligomeric IAPP (oIAPP) for 12 h, the HBVP displayed a round shape more frequently (Fig. 1C, D). The number of round HBVP per total number of HBVP was significantly higher compared to vehicle condition, which predominantly contained HBVP with an elongated morphology (Fig. 1C–E). To exclude that these morphological changes were due to apoptosis/necrosis, we stained with trypan blue, which specifically labels dead cells (Fig. 1F, G). We detected a very low proportion of trypan blue-positive round HBVP (less than 10%), and there were no significant differences between stimulations with oIAPP and vehicle (Fig. 1H).

### Validation of the microvascular model

**Fig. 2:**
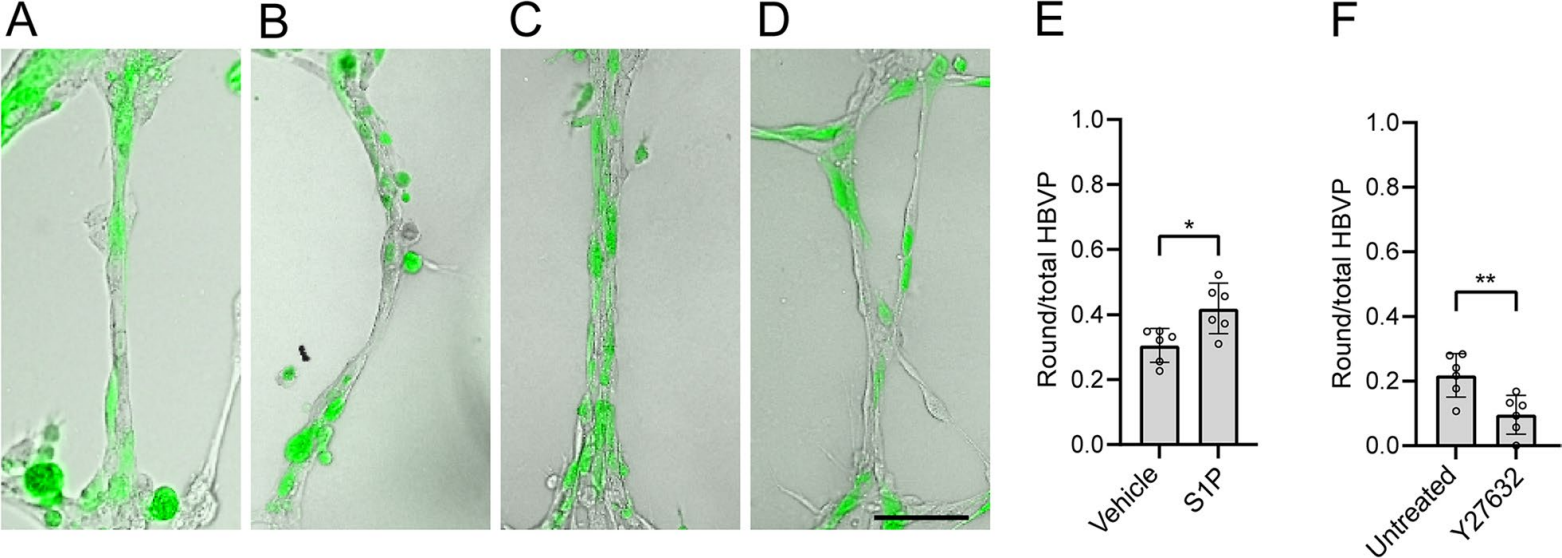
HBVP morphology in the microvascular model after stimulation with controls of contraction. Capillary-like structures (CLS) (HBVP: green) treated with vehicle **(A)**, with S1P **(B)**, untreated **(C)**, and treated with Y27632 **(D)** for 12 h, scale bar: 100 µm. Quantification of the proportion of round-shaped HBVP per CLS after stimulation is seen in **(E)** and **(F)**. All images were acquired with the 10 × objective. The experiments were performed in 6 replicates and data was analyzed using student t-test. Each point represent the mean ± SD. Significant difference at *p < 0.05. **p < 0.01

Since the oIAPP-induced changes in HBVP could not be explained by cell death, we investigated whether a round HBVP morphology is associated with contractile properties. Therefore, we analyzed the morphology after stimulating with the vasoconstrictor sphingosine 1-phosphate [22, 23], and the ROCK inhibitor Y27632, a vasodilator [24]. Compared with the vehicle condition (Fig. 2A), the stimulation with S1P (Fig. 2B) resulted in a significantly higher proportion of round HBVP, whereas after the treatment with Y27632 (Fig. 2D) this proportion was significantly lower (Fig. 2E, F).

### Stimulation with agonists and an antagonist of oIAPP

**Fig. 3:**
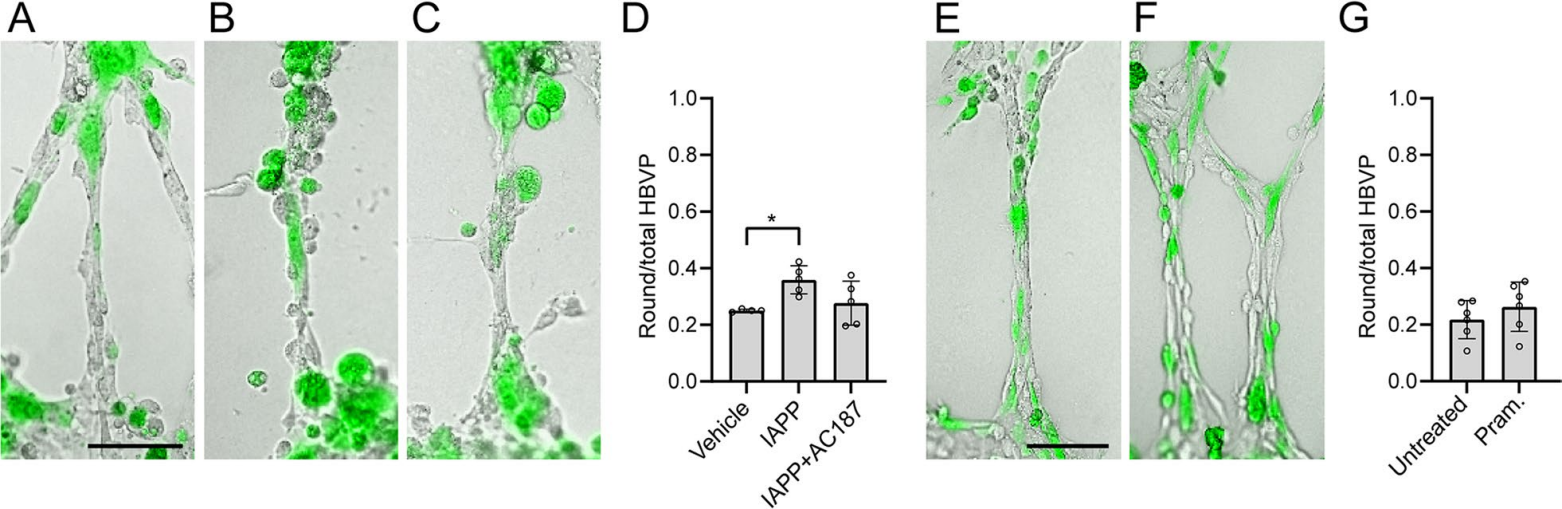
Effects of activation/inhibition of IAPP receptor. CLS after stimulation with vehicle **(A)**, IAPP **(B)**, and IAPP together with the antagonist AC187 **(C)**, scale bar **(A–C)**: 100 µm. Quantification of the proportion of round-shaped HBVP per CLS after stimulation with vehicle, IAPP, and IAPP with AC187 is seen in **(D)**. Untreated CLS **(E)** vs. after stimulation with the IAPP agonist pramlintide **(F)**, scale bar in **(E)** and **(F)**: 100 µm. Graph in **(G)** shows quantification of the proportion of round-shaped HBVP per CLS for untreated and pramlintide stimulation. All images were acquired with the 10 × objective. Experiments in **(D)** and **(G)** were performed in 6 replicates. In **(D)**, data was analyzed using one-way ANOVA followed by Dunnets correction test (n = 2 comparisons), in **(G)**, data was analyzed by student t-test. Each point represent the mean ± SD. Significant difference at *p < 0.05

Next, we aimed to investigate whether the impact of oIAPP on HBVP morphology is mediated via the IAPP receptor. We challenged the CLS with oIAPP together with the IAPP receptor antagonist AC187 [25] and found that the proportion of oIAPP-induced round HBVPs was no longer significantly higher compared to vehicle, but no significant difference was detected between oIAPP and oIAPP + AC187 treatments (0.359 ± 0.050 vs 0.277 ± 0.078, p = 0.094) (Fig. 3A–D). We also challenged our model with the IAPP receptor agonist pramlintide but found no alteration in the proportion of round HBVP (Fig. 3E–G).

### Stimulation with IAPP combined with Y27632, pramlintide, or blebbistatin

**Fig. 4:**
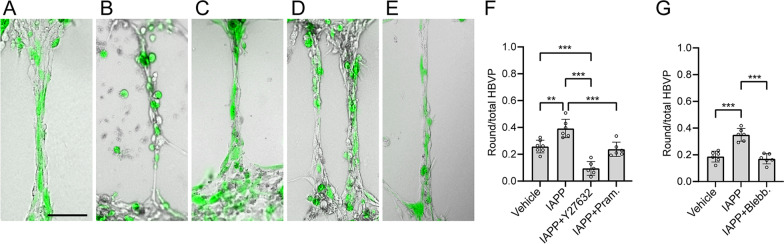
Treatments modifying the effect of oIAPP on HBVP. CLS after stimulation with vehicle **(A)**, oIAPP alone **(B)**, and oIAPP together with either Y27632 **(C)**, pramlintide **(D)** or blebbistatin **(E)**, scale bar **(A-E)**: 100 µm. Graphs in **(F)** and **(G)** show quantification of the proportion of round-shaped HBVP per CLS after stimulation. All images were acquired with the 10 × objective. Experiments in **(F)** and **(G)** were performed in 6 replicates and data was analyzed using one-way ANOVA followed by Tuckey test with (n = 6) comparisons **(F)** or (n = 3) comparisons **(G)**. Each point represent the mean ± SD. Significant difference at **p < 0.01, ***p < 0.001

To investigate the possibility to reverse the impact of oIAPP on HBVP morphology, we challenged the microvascular model with oIAPP together with either Y27632, pramlintide, or blebbistatin, an inhibitor of muscle and non-muscle myosin II [26] (Fig. 4A–E). All three molecules were able to reverse the impact of oIAPP, and the number of round HBVP was significantly lower in these conditions compared with the stimulation with only oIAPP (Fig. 4F, G). Supplementation with Y27632 together with oIAPP yielded additionally significantly lower number of round HBVP compared to vehicle.

### Quantification of vessel diameter in human brain tissue

**Fig. 5:**
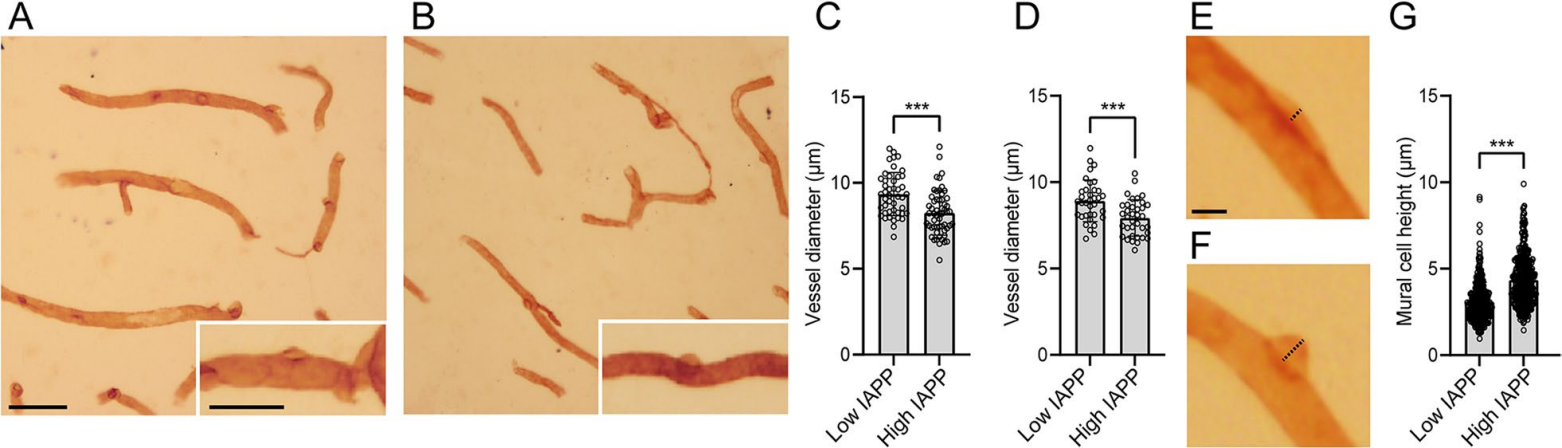
Analysis of capillary diameter in human tissue. Images in **(A)** and **(B)** show immunostainings for laminin in individuals with low **(A)** and high levels **(B)** of IAPP. Scale bars: 50 µm **(A)** and **(B)**, 25 µm (down to the right). Graph in **(C)** shows quantification of the diameter of laminin enclosed capillaries near laminin enclosed mural cells in individuals with low levels of IAPP (Low IAPP) and high levels of IAPP (High IAPP). Graph in **(D)** shows quantification of the diameter of laminin enclosed capillaries with no visible mural cells. Images in **(E)** and **(F)** show measurement of laminin enclosed mural cells with different heights were image in **(E)** shows a representative mural cell with low height and **(F)** represents a mural cell with high height. Scale bar: 5 µm **(E)** and **(F)**. Black dashed line in **(E)** and **(F)** indicate the measured distance. Graph in **(G)** shows quantification of the height of laminin enclosed mural cells in Low IAPP and High IAPP. Images in **(A)** and **(B)** were acquired with the 20 × objective, images in **(E)** and **(F)** were acquired with the 40 × objective. Data in **(C)**, **(D)** and **(G)** were analyzed using student t-test. Each point represents the diameter of one capillary in **(C)** and **(D)**. Each point represents one measurement of mural cell height in **(G)**. Significant difference at ***p < 0.001

Finally, to investigate the potential association between brain IAPP levels and mural cell contraction in clinical material, we analyzed the diameter of laminin-enclosed capillaries in the CA1 hippocampal region of neuropathologically evaluated individuals (n = 8). Levels of total IAPP in homogenates from this region of these individuals have been analyzed previously [19] and the individuals were divided into two groups: high (n = 4) and low (n = 4) IAPP levels. Table 1 shows gender, age, neuropathological assessment (ABC staging and Lewy Bodies), presence of T2DM, total IAPP levels, postmortem delay, and cause of death of the individuals included in the analysis. The diameter of laminin-enclosed capillaries near mural cells was significantly lower in individuals with high levels of total IAPP (Fig. 5C). The diameter of laminin-enclosed capillaries with no visible laminin-enclosed mural cells was also significantly lower in individuals with high total IAPP levels (Fig. 5D). To investigate whether the mural cell morphology is also affected in individuals with high levels of IAPP, we analyzed the height of the laminin-enclosed mural cell bodies (representative images in Fig. 5E, F). The analysis showed that individuals with high levels of IAPP showed significantly higher height compared to individuals with low levels of IAPP (Fig. 5G).

## Discussion

The aim of this study was to investigate the effect of oIAPP on HBVP morphology and contractility using an in vitro microvascular model. Using the microvascular model, we demonstrated that oIAPP cause changes in HBVP morphology, displaying a round shape more frequently. The morphological changes could not be explained by increased HBVP death, as the proportion of dead cells was very low compared with the total number of round HBVP and was not significantly increased by oIAPP stimulation. We, therefore, explored the impact of oIAPP on HBVP tone. The contractile properties of HBVP involve myosin regulatory light chain 9 [27], as well as the RhoA/ROCK pathway [28]. Whether oIAPP affects this pathway has not been described before, but Aβ oligomers, which share many properties with oIAPP [29], are known to both activate ROCK [30] and to induce pericyte contraction [31]. Hence, to investigate if HBVP morphological changes were due to contraction, we stimulated our model with the vasoconstrictor sphingosine 1-phosphate, an activator of the RhoA/ROCK pathway [22, 23], and the ROCK inhibitor Y27632, a vasodilator [24], alone and together with oIAPP. Indeed, S1P alone mimicked the effects of oIAPP on HBVP morphology, and both Y27632 and blebbistatin, a myosin inhibitor [26], reverted the effect of oIAPP on HBVP morphology. Of note, all three substances (S1P, blebbistation and Y27632) have been shown to affect endothelial cells in different ways. S1P promotes for example endothelial barrier protection [32, 33] and activates the endothelial RhoA/ROCK pathway [34], while altered endothelial calcium wave propagation [35] and endothelial cell basal tone and tension [36] have been found after blebbistatin stimulation. Y27632 has been shown to suppress endothelial RhoA/ROCK activation and improve endothelial cell viability [37] and in addition increasing the expression of the vascular endothelial (VE)-cadherin in cell-to-cell junctions [38]. In view of the known impact of these substances on endothelial cells, we can only assume that the endothelial cells in our model are also affected in our experiments. Hence, given the tight reciprocal communication between endothelial cells and pericytes [39], we cannot exclude the possibility that the found pericyte reaction (contraction or relaxation depending on substances) could partly be a result of an indirect pericyte response to endothelial cell reactions to the three substances. Nevertheless, our finding suggest that oIAPP, indirectly or directly, and just like Aβ, induce HBVP contraction, and that this induction might be mediated via the RhoA/ROCK pathway. To date there are no studies (to our knowledge) demonstrating a direct impact of oIAPP in the RhoA/ROCK pathway, but since oIAPP (just like Aβ oligomers) binds to the receptor for advanced glycation end products (RAGE) [40], and this receptor is in turn known to activate RhoA/ROCK pathway in different cell types (including retinal pericytes [41], microvascular pulmonary endothelial cells [42] and BV2 microglial cells [43]), we speculate that oIAPP induced the contraction in our experiments via RAGE.

The impact of IAPP on mural cell contraction in humans has not been reported before, but there are a few studies describing an effect of IAPP on the vascular tone in rats. These studies are, however, inconsistent. One study indicated that rat IAPP (rIAPP) induced relaxation of rat pulmonary arteries [44], while another study revealed reduced relaxation of rat mesenteric arteries after rIAPP treatment [45]. Of note, rodent IAPP is non-aggregative, and, hence, corresponds to the monomeric and more beneficial version of human IAPP. In addition, these experiments were performed in arteries, whose mural cells may respond to IAPP in a different way compared to the ones in capillaries. Our study, where we used a microvascular model to mimic human capillaries, demonstrate that oIAPP induces a key morphological hallmark of contraction in HBVP. These findings were further backed-up by our human postmortem study, where we analyzed the brain capillaries of individuals with high and low levels of IAPP. Our results show that individuals with high levels of total hippocampal IAPP display significantly smaller laminin-enclosed capillary diameters as well as a significantly lower number of laminin-enclosed mural cells with a flattened morphology in CA1. Of note, the shift from elongated flat morphology to a more triangular morphology has previously been described as an indication of pericyte migration [46]. Hence, although a number of studies support the idea that pericyte, in addition to smooth muscle cells, have contractile properties (for review see [47]), we cannot rule out that migration of pericytes in the analyzed individuals might have affected the results. Moreover, the ELISA used for analysis of hippocampal IAPP does not distinguish between aggregation forms, and hence our results do not reveal whether the reduced capillary diameter is related to oIAPP levels or higher amount of IAPP in general (i.e. monomers, oligomer or fibrils). It should further be emphazised that previous studies have shown an impact of Aβ on pericyte contraction [29], therefore, we selected cases with no or very scarce amyloid pathology in the hippocampus. However, most elderly individuals display some Aβ pathology, despite the lack of neurodegenerative changes linked to Alzheimer’s dementia or other dementia forms. Therefore, we cannot completely rule out the possibility that Aβ pathology, not captured by the neuropathological evaluation (such as Aβ oligomers), also has an impact on the analyzed capillary diameter. In addition, the number of cases included in the study was low (due to shortage of cases completely free from Aβ pathology), which is a limitation of the study. Nevertheless, taken together, our findings point towards a direct impact of IAPP on mural cells, inducing capillary contraction in humans.

Next, we investigated the role of IAPP receptor in the morphological changes observed in the HBVP in our model. The IAPP receptor (AMY) is a heterodimer consisting of the calcitonin receptor (CTR) and a receptor activity-modifying protein (RAMP) [48]. There are 6 isoforms of the receptor (AMY_1-3(a/b)_), formed by combinations of CTRa/CTRb with RAMP 1–3, and the distribution of the isoforms in different tissues is difficult to assess [1]. Interestingly, our results did not show a clear involvement of the receptor in oIAPP-induced HBVP contraction. Although the mean proportion of round HBVP after supplementation with the AMY receptor antagonist AC187 together with oIAPP did not significantly differ from vehicle, it did not either differ from the proportion of round HBVP after oIAPP stimulation alone. In addition, stimulation of the receptor with IAPP agonist pramlintide had no effect. This could be explained by the fact that different isoforms of the receptor have different affinities for oIAPP, AC187, and pramlintide. In addition, while it is known that the AMY receptor has an affinity for IAPP in its monomeric/non-aggregative form, little is known about its affinity for oIAPP. Hence, it is important to consider other pathways potentially influenced by oIAPP, such as membrane permeabilization, alternative receptors, or other mechanisms of cell transport. For example, human IAPP (hIAPP) oligomers are known to form pores, which lead to the disruption of membrane integrity and an increase in reactive oxygen species [49]. hIAPP can also form channels permeable for Na^+^, K^+^, Ca^2+^, and Cl^−^ in lipid bilayer membranes [50]. Whether the latter mechanism also occurs in mural cells has not been investigated yet, but if it does, it is tempting to speculate that this leads to increased intracellular concentration of Ca^2+^, which, in turn, would increase contraction and eventually lead to apoptosis. Also, other receptors beside the AMY receptor may be implicated. For example oIAPP also binds to RAGE [51, 52]. This receptor, which is found on pericytes [53, 54], is known to mediate the toxic effects induced by IAPP in pancreatic β-cells [51]. Hence, we cannot rule out the possibility that RAGE or other receptors with affinity for IAPP are also involved in the oIAPP-induced HBVP morphological changes shown in this study. Finally, oIAPP might also be internalized by mural cells via different mechanisms of cellular transport and thereby cause morphological changes. A support for this idea can be found in studies describing an uptake of oIAPP via translocation, micropinocytosis, and clathrin-mediated endocytosis in pancreatic β-cells [55]. All these possible mechanisms of oIAPP might act alone or combined, provoking the morphological changes we detected in the HBVP/mural cells.

Although the AMY receptor antagonist AC187 was unable to revert oIAPP-induced morphological changes in HBVP, we found that Y27632, blebbistatin, and pramlintide had a potent reversal effect. Both Y27632 and blebbistatin are inhibitors of contraction [24, 26], which supports the idea that oIAPP induces an increase in HBVP contraction. Regarding the mechanism of action of pramlintide, it might be explained by a physical interaction with IAPP since previous studies have shown that pramlintide inhibits hIAPP aggregation in vitro [56]. Another possible mechanism could be that pramlintide competes with oIAPP for the same receptors and given its non-aggregative nature, pramlintide binding elicits a counteracting effect on the HBVP. From this perspective it is noteworthy that pramlintide, an FDA approved drug used together with insulin for T2DM treatment, has well-described beneficial effects. Apart from mimicking the physiological effects of monomeric IAPP [57], pramlintide improves learning and memory function in diabetic rats [58], and reverts the depression of the long-term potentiation caused by Aβ and oIAPP in mouse brain slices [59]. Besides, pramlintide enhances the removal of Aβ from the brain over the BBB, increasing the trafficking of the low-density lipoprotein receptor-related protein 1 (LRP-1), a well-known Aβ clearance transporter, to the membrane of endothelial cells in the BBB [14]. Since pericytes play an important role in this LRP-1 mediated Aβ clearance [60], it is tempting to speculate that the beneficial impact of pramlintide on Aβ clearance is in part mediated via its impact on pericyte/mural cell functionality as seen in our study.

Finally, the model used in our study admit investigations on HBVP when interacting with endothelial cells, which is different from monolayered in vitro models. Such system is useful when investigating isolated events (such as oIAPP exposure), but it is important to point out that cell cultures can never replicate a biological system. Nevertheless, our findings point towards a contraction effect of oIAPP on pericytes, and that this contraction can be reverted by pramlintide and contraction inhibitors. The significance of these findings may be several-fold. First, given that mural cells, like pericytes, regulate basal capillary blood flow resistance in the brain [61, 62], vascular accumulation of aggregated IAPP, as seen in demented T2DM patients and patients with AD, may underly the reduced blood flow in these patients. Secondly, although our studies focus on the impact of IAPP on mural cells in the brain, it is likely that similar cells in the periphery and retina also respond to IAPP in a similar way, an idea important to consider in future research on vascular complications in T2DM (such as retinopathy and diabetic foot). Thirdly, the ameliorating effect of pramlintide on oIAPP-induced contraction highlights the potential use of pramlintide not only for glucose control, but also to prevent vascular complications. Hence, we conclude that the impact of oIAPP on pericyte contraction might be an event important to target.

## Data Availability

The dataset supporting the conclusions of this article is available via ProteomeXchange with the identifier PXD035048.

## Abbreviations

AD: Alzheimer’s disease
AMY: The IAPP receptor
BBB: Blood brain barrier
Blebb: Blebbistatin
CA1: Cornu Ammonis
CLS: Capillary-like structures
COPD: Chronic obstructive pulmonary disease
CSF: Cerebrospinal fluid
HBVP: Primary human brain vascular pericytes
hCMEC/D3: Human cerebral microvascular endothelial cells
IAPP: Islet amyloid polypeptide
LB: Lewy bodies
LC–MS/MS: Liquid chromatography–tandem mass spectrometry
LRP-1: Low-density lipoprotein receptor-related protein 1
oIAPP: Oligomeric IAPP
Pram: Pramlintide
RAGE: Receptor for advanced glycation end products
RAMP: Receptor activity-modifying protein
S1P: Sphingosine-1-phosphate
T2DM: Type 2 diabetes mellitus

## References

[1] Mathiesen DS, Lund A, Holst JJ, Knop FK, Lutz TA, Bagger JI. Amylin and calcitonin—physiology and pharmacology. Eur J Endocrinol [Internet]. 2022;1. 10.1530/EJE-21-1261 35353712

[2] Westermark P (1972). Quantitative studies of amyloid in the islets of langerhans. Ups J Med Sci [Internet]..

[3] Clark A, Wells CA, Buley ID, Cruickshank JK, Vanhegan RI, Matthews DR (1988). Islet amyloid, increased A-cells, reduced B-cells and exocrine fibrosis: quantitative changes in the pancreas in type 2 diabetes. Diabetes Res [Internet]..

[4] Jurgens CA, Toukatly MN, Fligner CL, Udayasankar J, Subramanian SL, Zraika S (2011). β-Cell loss and β-cell apoptosis in human type 2 diabetes are related to islet amyloid deposition. Am J Pathol [Internet]..

[5] Banks WA, Kastin AJ (1998). Differential permeability of the blood-brain barrier to two pancreatic peptides: Insulin and amylin. Peptides (NY) [Internet]..

[6] Banks WA, Kastin AJ, Maness LM, Huang W, Jaspan JB (1995). Permeability of the blood-brain barrier to amylin. Life Sci [Internet]..

[7] Jackson K, Barisone GA, Diaz E, Jin LW, DeCarli C, Despa F (2013). Amylin deposition in the brain: a second amyloid in Alzheimer disease?. Ann Neurol Ann Neurol.

[8] Martinez-Valbuena I, Valenti-Azcarate R, Amat-Villegas I, Riverol M, Marcilla I, Andrea CE (2019). Amylin as a potential link between type 2 diabetes and alzheimer disease. Ann Neurol [Internet]..

[9] Exalto LG, Biessels GJ, Karter AJ, Huang ES, Katon WJ, Minkoff JR (2013). Risk score for prediction of 10 year dementia risk in individuals with type 2 diabetes: a cohort study. Lancet Diabetes Endocrinol [Internet]..

[10] Ly H, Verma N, Wu F, Liu M, Saatman KE, Nelson PT (2017). Brain microvascular injury and white matter disease provoked by diabetes-associated hyperamylinemia. Ann Neurol.

[11] Schultz N, Byman E, Fex M, Wennström M (2017). Amylin alters human brain pericyte viability and NG2 expression. J Cerebral Blood Flow Metab [Internet]..

[12] Sweeney MD, Ayyadurai S, Zlokovic BV. Pericytes of the neurovascular unit: Key functions and signaling pathways. Nat Neurosci. 2016; 771–83. 10.1038/nn.4288 PMC5745011 27227366

[13] Gonzales AL, Klug NR, Moshkforoush A, Lee JC, Lee FK, Shui B (2020). Contractile pericytes determine the direction of blood flow at capillary junctions. Proc Natl Acad Sci U S A [Internet]..

[14] Mohamed LA, Zhu H, Mousa YM, Wang E, Qiu WQ, Kaddoumi A (2017). Amylin enhances amyloid-β peptide brain to blood efflux across the blood-brain barrier. J Alzheimer’s Dis [Internet]..

[15] Schultz N, Janelidze S, Byman E, Minthon L, Nägga K, Hansson O, et al. Levels of islet amyloid polypeptide in cerebrospinal fluid and plasma from patients with Alzheimer’s disease. PLoS One [Internet]. 2019;14. 10.1371/journal.pone.0218561 PMC6576764 31206565

[16] Brännström K, Öhman A, Nilsson L, Pihl M, Sandblad L, Olofsson A (2014). The N-terminal region of amyloid β controls the aggregation rate and fibril stability at low pH through a gain of function mechanism. J Am Chem Soc [Internet]..

[17] Montine TJ, Phelps CH, Beach TG, Bigio EH, Cairns NJ, Dickson DW (2012). National institute on aging-Alzheimer’s association guidelines for the neuropathologic assessment of Alzheimer’s disease: a practical approach. Acta Neuropathol.

[18] Braak H, del Tredici K, Rüb U, de Vos RAI, Jansen Steur ENH, Braak E (2003). Staging of brain pathology related to sporadic Parkinson’s disease. Neurobiol Aging.

[19] Schultz N, Byman E, Wennström M (2018). Levels of retinal IAPP are altered in Alzheimer’s disease patients and correlate with vascular changes and hippocampal IAPP levels. Neurobiol Aging [Internet]..

[20] Itoh Y, Toriumi H, Yamada S, Hoshino H, Suzuki N (2011). Astrocytes and pericytes cooperatively maintain a capillary-like structure composed of endothelial cells on gel matrix. Brain Res.

[21] Perrot CY, Herrera JL, Fournier-Goss AE, Komatsu M (2020). Prostaglandin E2 breaks down pericyte-endothelial cell interaction via EP1 and EP4-dependent downregulation of pericyte N-cadherin, connexin-43, and R-Ras. Sci Rep.

[22] Coussin F, Scott RH, Wise A, Nixon GF (2002). Comparison of sphingosine 1-phosphate-induced intracellular signaling pathways in vascular smooth muscles. Circ Res.

[23] Salomone S, Yoshimura SI, Reuter U, Foley M, Thomas SS, Moskowitz MA (2003). S1P3 receptors mediate the potent constriction of cerebral arteries by sphingosine-1-phosphate. Eur J Pharmacol Elsevier.

[24] Wang S, Cao C, Chen Z, Bankaitis V, Tzima E, Sheibani N (2012). Pericytes regulate vascular basement membrane remodeling and govern neutrophil extravasation during inflammation. PLoS One [Internet]..

[25] Young AA, Gedulin B, Gaeta LSL, Prickett KS, Beaumont K, Larson E (1994). Selective amylin antagonist suppresses rise in plasma lactate after intravenous glucose in the rat. Evidence for a metabolic role of endogenous amylin. FEBS Lett.

[26] Straight AF, Cheung A, Limouze J, Chen I, Westwood NJ, Sellers JR (1979). Dissecting temporal and spatial control of cytokinesis with a myosin II inhibitor. Science.

[27] He L, Vanlandewijck M, Raschperger E, Andaloussi Maë M, Jung B, Lebouvier T (2016). Analysis of the brain mural cell transcriptome. Sci Rep [Internet]..

[28] Kutcher ME, Kolyada AY, Surks HK, Herman IM (2007). Pericyte Rho GTPase mediates both pericyte contractile phenotype and capillary endothelial growth state. Am J Pathol [Internet]..

[29] O’Nuallain B, Williams AD, Westermark P, Wetzel R (2004). Seeding specificity in amyloid growth induced by heterologous fibrils. J Biol Chem.

[30] Henderson BW, Gentry EG, Rush T, Troncoso JC, Thambisetty M, Montine TJ (2016). Rho-associated protein kinase 1 (ROCK1) is increased in Alzheimer’s disease and ROCK1 depletion reduces amyloid-β levels in brain. J Neurochem [Internet]..

[31] Nortley R, Korte N, Izquierdo P, Hirunpattarasilp C, Mishra A, Jaunmuktane Z, et al. Amyloid b oligomers constrict human capillaries in Alzheimer’s disease via signaling to pericytes. Science (1979). 2019;365. 10.1126/science.aav9518. PMC6658218 31221773

[32] Reinhard NR, Mastop M, Yin T, Wu Y, Bosma EK, Gadella TWJ (2017). The balance between Gαi-Cdc42/Rac and Gα12/13-RhoA pathways determines endothelial barrier regulation by sphingosine-1-phosphate. Mol Biol Cell.

[33] Hansen L, Lohfink N, Vutukuri R, Kestner RI, Trautmann S, Hecht M (2022). Endothelial sphingosine-1-phosphate receptor 4 regulates blood-brain barrier permeability and promotes a homeostatic endothelial phenotype. J Neurosci.

[34] Liu W, Liu B, Liu S, Zhang J, Lin S (2016). Sphingosine-1-phosphate receptor 2 mediates endothelial cells dysfunction by PI3K-Akt pathway under high glucose condition. Eur J Pharmacol.

[35] Ponsaerts R, D’Hondt C, Bultynck G, Srinivas SP, Vereecke J, Himpens B (2008). The myosin II ATPase inhibitor blebbistatin prevents thrombin-induced inhibition of intercellular calcium wave propagation in corneal endothelial cells. Invest Ophthalmol Vis Sci.

[36] Goeckeler ZM, Bridgman PC, Wysolmerski RB. Nonmuscle myosin II is responsible for maintaining endothelial cell basal tone and stress fiber integrity. Am J Physiol Cell Physiol; 2008;295. 10.1152/ajpcell.00318.2008 PMC2575828 18701651

[37] Qi Y, Liang X, Hu X, He H, Tang L, Yao W. Tetrahydroxystilbene glucoside protects against LPS-induced endothelial dysfunction via inhibiting RhoA/ROCK signaling and F-actin remodeling. Gen Physiol Biophys; 2020;39:407–17. 10.4149/gpb_2020028 33084595

[38] Li X, Li X, Sun R, Gao M, Wang H. Cadmium exposure enhances VE-cadherin expression in endothelial cells via suppression of ROCK signaling. Exp Ther Med; 2022;23. 10.3892/etm.2022.11282 PMC9016791 35481222

[39] Armulik A, Abramsson A, Betsholtz C (2005). Endothelial/pericyte interactions. Circ Res [Internet]..

[40] Abedini A, Cao P, Plesner A, Zhang J, He M, Derk J (2018). RAGE binds preamyloid IAPP intermediates and mediates pancreatic β cell proteotoxicity. J Clin Invest.

[41] Zhang SS, Hu JQ, Liu XH, Chen LX, Chen H, Guo XH, et al. Role of moesin phosphorylation in retinal pericyte migration and detachment induced by advanced glycation endproducts. Front Endocrinol (Lausanne); 2020;11. 10.3389/fendo.2020.603450 PMC7708375 33312163

[42] Zhao MJ, Jiang HR, Sun JW, Wang ZA, Hu B, Zhu CR, et al. Roles of RAGE/ROCK1 pathway in HMGB1-induced early changes in barrier permeability of human pulmonary microvascular endothelial cell. Front Immunol; 2021;12. 10.3389/fimmu.2021.697071 PMC8564108 34745088

[43] Chen J, Sun Z, Jin M, Tu Y, Wang S, Yang X (2017). Inhibition of AGEs/RAGE/Rho/ROCK pathway suppresses non-specific neuroinflammation by regulating BV2 microglial M1/M2 polarization through the NF-κB pathway. J Neuroimmunol.

[44] Golpon HA, Puechner A, Welte T, Wichert PV, Feddersen CO (2001). Vasorelaxant effect of glucagon-like peptide-(7–36)amide and amylin on the pulmonary circulation of the rat. Regul Pept.

[45] el Assar M, Angulo J, Santos-Ruiz M, Moreno P, Novials A, Villanueva-Peñacarrillo ML, et al. Differential effect of amylin on endothelial-dependent vasodilation in mesenteric arteries from control and insulin resistant rats. PLoS One. 2015;10. 10.1371/journal.pone.0120479 PMC4373784 25807378

[46] Pfister F, Feng Y, vom Hagen F, Hoffmann S, Molema G, Hillebrands JL (2008). Pericyte migration: a novel mechanism of pericyte loss in experimental diabetic retinopathy. Diabetes [Internet]..

[47] Hamilton NB. Pericyte-mediated regulation of capillary diameter: a component of neurovascular coupling in health and disease. Front Neuroenergetics [Internet]. 2010;2. 10.3389/fnene.2010.00005 PMC2912025 20725515

[48] Christopoulos G, Perry KJ, Morfis M, Tilakaratne N, Gao Y, Fraser NJ (1999). Multiple amylin receptors arise from receptor activity-modifying protein interaction with the calcitonin receptor gene product. Mol Pharmacol [Internet]..

[49] Zhang N, Xing Y, Yu Y, Liu C, Jin B, Huo L (2020). Influence of human amylin on the membrane stability of rat primary hippocampal neurons. Aging [Internet]..

[50] Mirzabekov TA, Lin MC, Kagan BL (1996). Pore formation by the cytotoxic islet amyloid peptide amylin. J Biol Chem.

[51] Abedini A, Cao P, Plesner A, Zhang J, He M, Derk J (2018). RAGE binds preamyloid IAPP intermediates and mediates pancreatic β cell proteotoxicity. J Clin Invest [Internet]..

[52] Sturchler E, Galichet A, Weibel M, Leclerc E, Heizmann CW (2008). Site-specific blockade of RAGE-Vd prevents amyloid-β oligomer neurotoxicity. J Neurosci [Internet]..

[53] Yonekura H, Yamamoto Y, Sakurai S, Petrova RG, Joynal ABEDIN M, Yasui K, et al. Novel splice variants of the receptor for advanced glycation end-products expressed in human vascular endothelial cells and pericytes, and their putative roles in diabetes-induced vascular injury. Biochem J. 2003. 10.1042/BJ20021371 PMC1223244 12495433

[54] Kim J, Kim CS, Sohn E, Kim JS (2016). Cytoplasmic translocation of high-mobility group box-1 protein is induced by diabetes and high glucose in retinal pericytes. Mol Med Rep [Internet]..

[55] Trikha S, Jeremic AM. Distinct internalization pathways of human amylin monomers and its cytotoxic oligomers in pancreatic cells. PLoS One. 2013;8. 10.1371/journal.pone.0073080 PMC3760900 24019897

[56] Wang H, Ridgway Z, Cao P, Ruzsicska B, Raleigh DP (2015). Analysis of the ability of pramlintide to inhibit amyloid formation by human islet amyloid polypeptide reveals a balance between optimal recognition and reduced amyloidogenicity. Biochemistry [Internet]..

[57] Kruger DF, Gloster MA (2004). Pramlintide for the treatment of insulin-requiring diabetes mellitus: rationale and review of clinical data [Internet]. Drugs.

[58] Nassar SZ, Badae NM, Issa YA (2020). Effect of amylin on memory and central insulin resistance in a rat model of Alzheimer’s disease. Arch Physiol Biochem [Internet]..

[59] Kimura R, MacTavish D, Yang J, Westaway D, Jhamandas JH (2017). Pramlintide antagonizes beta amyloid (Aβ)- and human amylin-induced depression of hippocampal long-term potentiation. Mol Neurobiol [Internet].

[60] Ma Q, Zhao Z, Sagare AP, Wu Y, Wang M, Owens NC, et al. Blood-brain barrier-associated pericytes internalize and clear aggregated amyloid-β42 by LRP1-dependent apolipoprotein e isoform-specific mechanism. Mol Neurodegener [Internet]. 2018;13. 10.1186/s13024-018-0286-0 PMC6194676 30340601

[61] Hartmann DA, Coelho-Santos V, Shih AY (2021). Pericyte control of blood flow across microvascular zones in the central nervous system. Annu Rev Physiol [Internet]..

[62] Hartmann DA, Berthiaume AA, Grant RI, Harrill SA, Koski T, Tieu T (2021). Brain capillary pericytes exert a substantial but slow influence on blood flow. Nat Neurosci [Internet]..

